# Robotic vs. open surgery in obese women with low-grade endometrial cancer: comparison of costs and quality of life measures

**DOI:** 10.1186/s13584-020-00412-2

**Published:** 2020-11-02

**Authors:** Adi Sofer, Racheli Magnezi, Ram Eitan, Oded Raban, Orna Tal, Noam Smorgic, Zvi Vaknin

**Affiliations:** 1 Department of Management, Bar Ilan University, Ramat Gan, Israel; 2 Department of Obstetrics and Gynecology, Helen Schneider Hospital for Women, Rabin Medical Center–Beilinson Hospital, Petach Tikva, Israel; 3 Sackler School of Medicine, Tel Aviv University, Tel Aviv, Israel; 4 Yitzhak Shamir (formerly Assaf Harofeh) Medical Center, Zerifin, Israel; 5 Department of Obstetrics and Gynecology, Yitzhak Shamir (formerly Assaf Harofeh) Medical Center, 70300 Zerifin, Israel

**Keywords:** Endometrial cancer, Obese women, Robotic surgery, Costs, Quality of life, Minimally invasive surgery

## Abstract

**Background:**

This retrospective study compared perioperative measures, costs, quality of life and survival after open vs. robotic surgery, among obese women diagnosed with low-grade endometrial cancer.

**Methods:**

Obese women (body mass index (BMI) ≥ 30) who underwent open or robotic surgery for endometrial cancer, in one of two tertiary medical centers in the center of Israel, 2013–2016, postoperative grade 1–2, were included. Costs per patient, including 30-days post-surgery were calculated. Quality of life was evaluated by Physical and Mental Components of the SF-36 and a recovery from surgery questionnaire. Overall survival outcomes were obtained from patients’ files. Surgical outcomes, including operating and anesthesia times, length of hospital stay, and intraoperative and postoperative complications according to the Clavien-Dindo classification scale were reviewed.

**Results:**

In all, 138 women with BMI ≥30 underwent open (*n* = 61) or robotic surgery (*n* = 77) during the study period. The groups had similar BMI, comorbidities, demographics and tumor characteristics. Robotic surgery was associated with shorter hospital stays (mean 1.7 vs. 4.8 days; *P* < .0001) and fewer postoperative complications (Clavien-Dindo > 2, 5.2% vs. 19.7%; *P* = .0008), but longer operating theater time (3.8 vs. 2.8 h; *P* < .001). Costs are equivalent when at least 350 robotic surgeries are performed annually, not including the initial system costs. Quality of life measures were better after robotic surgery. SF-36 showed better measures for robotic surgery (Physical 56 vs. 39 and Mental 73 vs. 56; *P* < .01). After robotic surgery, patients tended to recover quicker when compared to open surgery, as they returned to normal activities earlier, with less need for family and governmental assistance (mean recovery time, 23 vs. 70 days; *P* < 0.006 and mean change in preoperative total functioning score, − 1.5 vs. -3.9: *P* < 0.05, respectively). Overall, 5-year survival was 89.8% for the open surgery group vs. 94% for the robotic surgery group (log rank, *P* = 0.330).

**Conclusions:**

Obese women with low-grade endometrial cancer had better quality of life after robotic vs. open surgery. They also had shorter hospital stays and fewer postoperative complications. Centers with high volumes of robotic surgery can achieve similar costs when comparing both methods. These results were achieved without jeopardizing survival. Our results further emphasize the need for the Israeli healthcare system to include specific reimbursement for robotic procedures in the population we studied.

## Introduction

Endometrial cancer (EC) is the most common gynecological malignancy in developed countries [1]. Often, women who develop EC are obese and have comorbidities such as hypertension and diabetes mellitus. Surgery is the first and most effective treatment and open surgery has been the traditional option. A minimally invasive approach (i.e. laparoscopy) was shown to be more efficient in terms of fewer perioperative complications and faster recovery [2], without compromising survival [3, 4]. Yet, studies showed that the conversion rate from laparoscopy to open surgery is related to obesity and age, reaching over 40% for women with body mass index (BMI) > 30 and older than 63-years-of-age [3]. The rate of robotic surgery (RS) in these studies was negligible, while other studies [5, 6] focusing on RS, reported that women with high BMI had lower conversion rates [7].

The robotic platform in gyneco-oncology was first introduced to public medical centers in Israel in 2009 and was slowly adopted by others throughout the country. The current study is the first to provide data regarding costs and quality of life related to RS in the Israeli population.

This study compared perioperative measures, costs, quality of life and survival among obese women diagnosed with low-grade endometrial cancer, who underwent open or robotic surgery in two tertiary medical centers in central Israel.

## Methods

This retrospective, observational study was conducted in two tertiary medical centers in central Israel where RS was performed. All consecutive women with BMI ≥30, undergoing surgery for low-grade endometrial cancer (postoperative grade 1–2), during 2013–2016 were included.

In both medical centers, the decision to perform robotic versus open surgery, was primarily based on the availability of the robotic system. In Yitzhak Shamir (formerly Assaf Harofeh) Medical Center, it was limited to 1–2 gynecologic cases per month. In Rabin Medical Center (Beilinson Hospital), it was limited to 2–3 gynecologic cases per month during the study period.

Surgeons recommended RS for all eligible women. If it was not available, they were given the option to get on the waiting list or undergo open surgery. The availability was limited by the budgets of the medical centers.

The women underwent open or RS, that included acquisition of peritoneal fluid or washings for cytology, and total hysterectomy, bilateral salpingo-oophorectomy. Pelvic and Para-aortic lymph-nodes were sampled or dissected according to risk classification, based on the surgeon’s decision.

Data abstracted from the electronic medical records included age, BMI, comorbidities, type of surgical technique and conversion rate. Operation time (OR) was defined as the total time the patient was in the operating theater. Procedure time was defined as the time from skin incision to skin closure (STS). The duration of hospitalization was defined as the period from the day of the procedure to the day of discharge.

Readmissions and surgical complications (such as wound infections, urinary or pulmonary tract infections, need for blood transfusion, etc.) within 30 days were also collected and classified according to the Clavien-Dindo scale [8]. The scale includes 5 grades of surgical complications. Grade I includes any change from the normal post-operative course without the need for intervention. Grade II are complications includes blood transfusion and pharmacological intervention. Grade III includes surgical, endoscopic or radiological interventions. Grade IV are life-threatening complications requiring intensive care and Grade V is death. The letter d is added to the grade if a patient is discharged with a disability that requires follow-up.

Tumor stage was classified according to the International Federation of Gynecology and Obstetrics (FIGO) classification system [9]. This system includes 4 main stages for cancer of the endometrium. In Stage I, the cancer is confined to the corpus uteri, whereas in Stage IV the cancer has spread to adjacent pelvic (Stage IVA) or distant (Stage IVB) organs.

The costs of the medical procedures were calculated according to the Cost per Treatment (CPT) system in Israel. In CPT procedures, such as hysterectomy, the price is fixed regardless of the technology used and covers 5 days of hospitalization. The costs in this study were calculated using hospital data about cost per day of hospitalization (e.g., laboratory, medications and staff) and operating theater per hour (including surgical instruments and disposables). The initial cost of the RS system assumed a 7-year depreciation, cost of yearly maintenance and an annual case load of 100 to 350 procedures/system. Costs in new Israeli shekels (NIS) were converted to US dollars (USD) using the 2016 mean currency rate ($1 = 3.84 NIS).

### Questionnaires

The study included 2 questionnaires. The SF-36 is a validated quality of life questionnaire that includes 36 questions with both physical and mental components. We used the validated Hebrew version [10]. The SF-36 is useful for surveying health in general populations, comparing the relative burden of diseases and benefits produced by treatments. It has 8-scales (Physical functioning, Role physical, Bodily pain, General health, Vitality, Social functioning, Role emotional and Mental health), each answer earns a score according the scoring method. Each of the 8 scales is measured by the average score of selected questions, and is transformed into a 0–100 scale. Due to variations in physical and mental health, the 8 scales are summarized and aggregated into a Physical Component Summary and Mental Component Summary (PCS, MCS). Each component is scored 0 to 100, where 0 is equivalent to maximum disability and 100 is equivalent to no disability [11]. In this study, we used the results of the PCS and MCS only.

A general questionnaire was developed to collect demographic data and to estimate patients’ recovery from surgery. This questionnaire was administered to four patients to check reliability and the time required to answer the questions. These four patients did not participate in the study. Their responses led to a minor adjustment in the question that dealt with the need for assistance with daily activities, termed self-treatment (ST). The questionnaire included 6 questions about the need for assistance with daily activities ST, daily functioning (DF) and mobility before and 4 weeks after the surgery. Each item was scored 1–4, (where 4 is totally independent and 1 is unable to perform). Change was defined as the difference between the scores after and before surgery. The difference in scores was calculated for each component (ST, DF and mobility). Women were also asked about the duration of time (in days) until they returned to their normal, daily activities, as defined before surgery.

Both questionnaires were answered, retrospectively on the phone, with no defined time period after the surgery.

### Data analysis

Medical and demographic characteristics were compared using the student *t* test, χ^2^ test and Mann-Whitney test, each as appropriate. General linear model and reliability statistics were used. The analysis was performed using SPSS software (version 23).

## Results

Of the 138 women who met the study criteria, 77 underwent RS and 61 open surgeries. There were no significant differences between the two groups regarding age, BMI, comorbidities, history of cancer or FIGO stage (Table 1). Surgical outcomes are shown in Table 2. The duration of procedures (STS) and total time in the operating theater were both longer for RS. Women who underwent RS had shorter hospitalization after surgery, and fewer postoperative complications based on the Clavien-Dindo classification scale.

**Table 1:** Patient and tumor characteristics

Variable	Laparotomy (*n* = 61)	Robotic surgery (*n* = 77)	*p*
Age, mean (range), years	65.4 (34–89)	62.6 (39–86)	0.104*
BMI, mean (range), kg/m^2^	36.48 (30–55)	36.62 (30–51)	0.454*
Preoperative Grade, n (%)			
EEC Grade 1	25 (41)	39 (51)	
EEC Grade 2	24 (39)	23 (30)	
EEC Grade 3	3 (5)	4(5)	
Uterine papillary serous carcinoma	2 (3)	0	
Clear cell cancer	2 (3)	0	
Complex hyperplasia with atypia	5 (8)	11 (14)	
Comorbidity, n (%)			0.617**
None	16 (26.2)	21 (27.3)	
Hypertension	16 (26.2)	24 (31.2)	
Diabetes	4 (6.6)	8 (10.4)	
Hypertension and diabetes	25 (41.0)	24 (31.2)	
History of other cancers			0.128**
None	56 (93.3)	66 (86.8)	
Breast cancer	4 (6.7)	5 (6.6)	
Other	0 (0.0)	5 (6.6)	

*Mann Whitney test, ^**^ χ^2^ test, as appropriate

**Table 2:** Surgical outcomes

Variable	Laparotomy (*n* = 61)	Robotic surgery (*n* = 77)	*p*
In Operating Room (anesthesia and surgery), min			<.001*
Mean	162	228	
Range	78–270	120–438	
Skin to skin, min			<.001*
Mean	126	174	
Range	54–234	72–408	
Hospitalization (after surgery), days			0.001*
Mean	4.8	1.7	
Range	2–23	1.0–4.0	
Postoperative complication			0.002**
Yes	19 (31.1)	8 (10.4)	
No	42 (68.9)	69 (89.6)	
Clavien-Dindo, n (%)			0.008**
None or I	49 (80.3)	73 (94.8)	
II or above	12 (19.7)	4 (5.2)	
Final Histology n (%)			0.581**
Grade I	20 (32.8)	28 (37.3)	
Grade II	41 (67.2)	47 (62.7)	
FIGO stage, n (%)			0.45**
I A	45 (78.9)	59 (78.7)	
I B	8 (14.0)	13 (17.3)	
II	2 (3.5)	2 (2.7)	
III A	2 (3.5)	0 (0.0)	
IV A	0 (0.0)	1 (1.3)	

*Mann Whitney test, ^**^χ^2^ test, as appropriate

The detailed complications include:

In the RS group – 4 patients with Clavien-Dindo level II complications - one with urinary tract infection, one with fever and diarrhea, one with fever of unknown source and one with wound infection - all were treated by antibiotics with no further complications.

In the open surgery group – 12 patients had level II – IV complications per Clavien-Dindo classification scale. Seven had grade II complications: 2 had wound infection, 3 had lower respiratory infection and 2 needed blood transfusion. Three patients had grade III-IV complications: all had wound complications with infection and needed surgical intervention.

Costs related to the type of procedure are summarized in Table 3. The costs were calculated based on the hospitals’ records. There were no significant differences in the costs of the surgical procedures and the related costs attributed to complications, when the initial and maintenance costs of the robotic system were not included. When we included the robotic maintenance costs, a volume of 350 cases per year was needed to break even. RS cost significantly more when the initial cost of the equipment was added to the equation.

**Table 3:** Estimated mean cost* per patient undergoing laparotomy or robotic surgery

Variable	Laparotomy (*n* = 61)	Robotic Surgery (*n* = 77)	*p*-value
CPP**			
Mean	8270	8850	0.148***
SD	20,598	6397	
CPP including *robotic maintenance*			
Mean	8270	10,850^$^/ 9422°	0.001/ 0.11**
SD	5363	1665	
CPP including *robotic maintenance and initial cost*			
Mean	8270	14,422^$^/10,442°	< 0.001/ 0.003**
SD	5363	1665	

*All prices are in US dollars. Exchange rate mean 2016: 1 USD = 3.84 NIS

**CPP-cost per patient, including all costs related to activities performed in operating room (surgical instruments), cost of 1 OR hour + recovery = 3600 NIS ($937) postoperative care including hospitalization, returning hospitalizations and ward costs (the cost per day of hospitalization = 3802 NIS ($990) and 30-days postoperative care). Annual robotic maintenance was 200,000 USD. Initial robotic system cost 2.5 million USD with assumed 7-year depreciation

***Student *t*-test

^$^Per 100 procedures per robotic system

°Per 350 procedures per robotic system

### Quality of life

The response rate for both questionnaires was 80/138 women (58%), 25 women (18%) were lost to follow-up and 33 (24%) refused to participate. The average time for the phone interview was about 30 min.

There were no statistical differences in age, BMI or comorbidities between the patients who answered the questionnaires and those who did not. Comparing the subgroups of women who answered the questionnaires with those refused to answer or who were lost to follow-up, also showed no significant differences in BMI (*F*(2,134) = 2.294, *ns*), age (*F*(2,134) = 2.227, *ns*) or background comorbidities (*χ*^*2*^(6) = 0.581, ns), Table 4.

**Table 4:** Comparison of participants regarding QOL questionnaires

Variable	Answered QOL	Refused	Lost to follow-up	*p*-value
BMI, mean, kg/m^2^	35.5	35.2	38.4	0.105
Age, mean, years	63.0	63.2	67.6	0.11
Comorbidities, n				
None	23	10	4	Χ^2^(6) = 0.581, ns (0.445)
Diabetes	3	6	3	
Hypertension	24	7	9	
Hypertension + Diabetes	30	10	9	

Comparisons of BMI, age and background co-morbidities among the subgroups regarding QOL questionnaires showed no significant differences

**Fig. 1:**
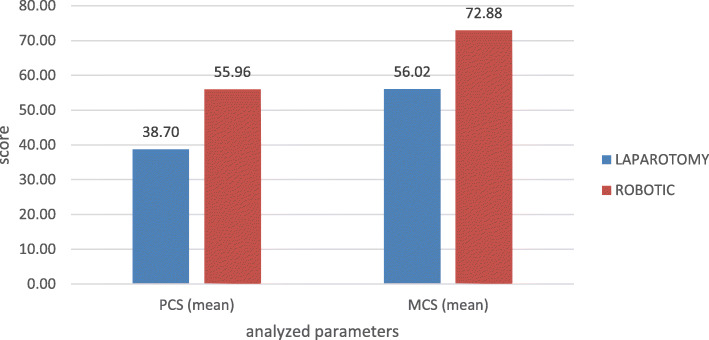
SF-36, comparison of physical component summary (PCS) and mental component summary (MCS) scores between patients who underwent laparotomy vs. robotic surgery (*P* < .01)

Patients who underwent RS scored higher in both the physical and mental components of the SF-36 (Fig. 1). For the PCS, RS was 56 vs. 38.7 for open surgery and 72.9 vs. 56, respectively for the MCS (*P* < .01, for both).

There were no differences between groups in the general questionnaire scores regarding specific daily activities before surgery, (ST, DF and mobility). The overall negative change regarding ST, DF and mobility parameters was smaller post-operatively for patients who had RS as compared to open surgery (− 1.51 vs. -3.88, respectively, *P* < .05). The RS group returned to daily activities sooner than the laparotomy group did (22.6 days vs. 70.4 days, respectively; *P* < 0.006).

### Overall survival

**Fig. 2:**
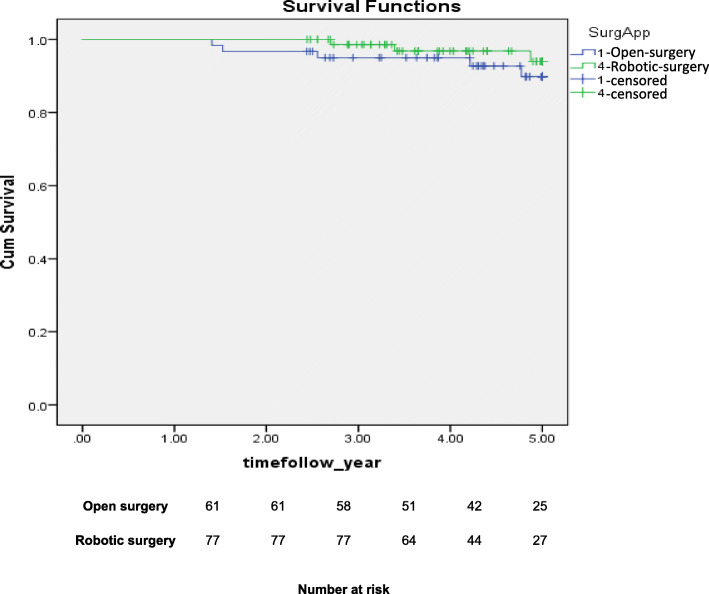
Overall survival of women with low grade endometrial cancer and BMI ≥30, open versus robotic surgery. In the open surgery group, overall survival was 89.8% and in the RS group, 94.0%, Log-rank *P* < 0.330. The Kaplan-Meier method was used

We found no significant differences between the groups regarding 5-year survival rates. The rates were 94.0% (95% CI 88.7–99.3) in the RS group vs. 89.8% in the open-surgery group ((95% CI 82.2–97.4); *P* = 0.330; Fig. 2).

## Discussion

This study reports costs and quality of life among obese women with low-grade endometrial cancer, who underwent robotic or open surgery. Although there are a few studies regarding these subjects from other countries, it is difficult to fully extrapolate their findings to the Israeli population and healthcare system. Our perioperative surgical outcomes agree with those reported by other investigators [12–16]. As in other studies, women undergoing RS for endometrial cancer were hospitalized for shorter periods than after open surgery; in the current study, 1.7 days vs. 4.8 days. These are in the same range reported by others, i.e. 1–3 days on average for RS, as compared to 4–8 days for open surgery [12–14].

We used the Clavien-Dindo scale to measure complications. The complication rate after RS was lower (5.2% vs 19.7%; *P* = 0.008) with no RS patients with grade III or more complication (versus 8.2% open surgery patients who had grade III-IV complications). Other studies [7, 17–19] also found better peri-operative outcomes, which reinforces the assertion that RS in obese patients is safer and more beneficial, as compared to laparotomy. As in our study, RS did not negatively affect survival [20].

These better outcomes were accompanied by longer operative procedure (STS) and longer total operating theater time in our study, as in other reports. In our study, the mean STS was 126 min for open surgery, as compared to 174 min for RS; similar to the reports by Subramaniam et al. [17] (138 min versus 246 min). The same was true for OR time: in our study 162 min for open surgery vs. 228 min for RS, similar to the reports by Subramaniam et al. [17] (191 vs. 303 min).

The major concern regarding the use the robotic Da Vinci platform is cost. All healthcare systems in the world struggle to lower costs while maintaining good, reasonable and achievable medical care. Costs are based on multiple factors that can vary from country to country based on the cost of living and type of healthcare system. The major variables that increase the costs of RS are the initial expenditure ($2.5 million in Israel), the on-going maintenance costs ($200,000 per year in Israel) and to some extent, the longer operative time ($937 per hour in Israel). Other costs include the disposable equipment per surgery, and costs of complications (calculated up to 30 days postoperative). Shorter hospital stays ($990 per day) are a cost savings. Comparing the costs of RS with the use of disposable equipment and yearly maintenance costs, averaged according to 350 procedures a year, found no significant difference in cost, when compared to laparotomy. Recent research conducted in Sweden [21] presented very similar costs between these two procedures (robotic € 10,683 and laparotomy € 11,073, *P* = 0.367) for the same type of cancer. Another study [22] with a sample of 120 patients, showed that healthcare costs for robotic-assisted laparoscopic surgery were significantly lower (€15,581 vs. €16,807, *P* < 0.05) than for laparotomy. Both studies [21, 22] included costs of the robotic system.

As the mortality rate for low-grade endometrial cancer is low, post-operative quality of life is important. This issue is the basis for the minimally invasive surgical approach. Our study found an advantage of RS over laparotomy in all quality of life measures evaluated. We also reported shorter time needed to “return to normal activity” in the RS group, as compared to the laparotomy group (22.6 days vs. 70.4 days, respectively). Our findings agree with those of a 2008 study [13] that compared laparotomy, laparoscopy and RS, focusing on “return to normal activity” at the time of follow-up examination and whether the patient had returned to work or to normal activities. The average time for patients who underwent RS to return to normal activity was significantly shorter than after laparotomy (24.1 days vs. 52 days, respectively; *p* < 0.0001).

Recovery time has a direct effect on a country’s economy. As of 2016, daily cost per employee (5 days a week) was $57.80 [23]. Women who take longer sick leaves cost more. Moreover, relatives may have to miss work to care for them. In our study, we tried to evaluate this parameter and include it in the total cost of each procedure. About 70% of women who answered the quality of life questionnaires did not work outside the home or were employed part-time. Therefore, we could not derive a full cost estimation that would reflect a complete picture. Nonetheless, we must consider that these women are still managing an independent lifestyle and household; therefore, their lack of independence costs money.

The current study had a few limitations. One weakness of this retrospective study is the inability to trace and determine the motives of each decision that was made by several physicians when a new patient needed treatment. Decisions regarding type of procedure were based on availability of equipment, staff, operating theaters and the patient’s medical status. As patients were not randomly allocated to each procedure, we cannot rule out the possibility of selection bias. In a case of selection bias, one group might include patients at greater risk, which can lead to higher rate of complications or poorer overall outcomes in that group. Yet, there were no statistical differences between the groups regarding the patients` characteristics, which lower the likelihood of a significant effect of such a bias on the results. Furthermore, our results are consistent with those of other studies [7, 17–19] that also support this point. However, if the patients who underwent robotic surgery were healthier or had more family support in ways not covered by the observed control variables, as compared to the patients who underwent open surgery, then the study might be overstating the advantages of robotic surgery, somewhat.

Women did not answer the questionnaires within a defined time frame. Responses were obtained 1.5 to 60 months after the procedure, with a mean of 34 months and a median of 36 months. The assumption was that women undergoing the surgery would remember it and the after-effects, but recall bias is possible.

Surgery using an advanced robotic system is more expensive than open surgery because it includes the costs of disposable equipment, ongoing maintenance and high initial expenditures. These parameters limit the widespread use of this platform in Israel, as in many other countries. In Israel, hospitals are not reimbursed for these procedures as a reflection of the actual costs of the technology, but at a fixed cost, equal to 5 days of hospitalization [24]. For RS to become more popular, reimbursement needs to be revised to include the actual costs of the new technology, such as with a Diagnosis-Related Group system.

## Conclusions

Obese women with low-grade endometrial cancer have better short-term quality of life after robotic surgery when compared to open surgery. They also benefit from shorter hospital stays and fewer postoperative complications. These results were achieved without jeopardizing survival. Centers with high volumes of robotic surgery can attain equivalent costs when both methods are compared. These results further emphasize the need for the Israeli healthcare system to include specific reimbursement for robotic procedures in the population we studied.

## Data Availability

Data can be made available upon reasonable request to the corresponding author.
